# Assessment of Interlaboratory Variation in the Interpretation of Genomic Test Results in Patients With Epilepsy

**DOI:** 10.1001/jamanetworkopen.2020.3812

**Published:** 2020-04-29

**Authors:** Jeffrey A. SoRelle, Juan M. Pascual, Garrett Gotway, Jason Y. Park

**Affiliations:** 1 Department of Pathology, University of Texas Southwestern Medical Center, Dallas; 2 Department of Pediatrics, University of Texas Southwestern Medical Center, Dallas; 3 Department of Neurology and Neurotherapeutics, University of Texas Southwestern Medical Center, Dallas; 4 Eugene McDermott Center for Human Growth and Development, University of Texas Southwestern Medical Center, Dallas; 5 Department of Internal Medicine, University of Texas Southwestern Medical Center, Dallas

## Abstract

**Importance:**

Discordance in the interpretations of genetic test results has occurred with the increased number of laboratories that are performing testing. Differences in diagnostic interpretations may have implications for the treatment of patients.

**Objective:**

To assess the interlaboratory variation in the interpretations of genetic test results with potential therapeutic implications.

**Design, Setting, and Participants:**

In this cross-sectional study, 70 genes that are commonly tested in patients with epilepsy were examined to identify 22 676 genetic variants from an unknown number of patients using the ClinVar public database of clinically annotated variants. Variant annotations submitted to ClinVar (data set version 2019-05) between November 16, 2012, and May 3, 2019, were included in the analysis. Conflicting interpretations of the genetic variants associated with epilepsy were analyzed for clinically substantial discrepancies between May 7 and June 29, 2019. Variants were examined only if they had been interpreted by 2 or more clinical laboratories. A variant with a clinically substantial difference in interpretation was defined as a variant that crossed the threshold between a likely pathogenic variant and a variant of uncertain significance.

**Main Outcomes and Measures:**

The frequency and types of variant interpretation conflicts were analyzed when a conflict was identified.

**Results:**

A total of 6292 of 22 676 variants related to epilepsy (27.7%) were interpreted by 2 or more clinical laboratories. Many variants (3307 of 6292 [52.6%]) had interpretations that were fully concordant. However, 2985 variants (47.4%) had conflicting interpretations. A clinically substantial conflict was identified in 201 of 6292 variants (3.2%). Furthermore, 117 of 201 variants (58.2%) with differences in interpretation occurred in genes with therapeutic implications.

**Conclusions and Relevance:**

In this cross-sectional study, most interpretations of genetic variants associated with epilepsy were concordant among laboratories, but more than half of the variants with conflicting interpretations occurred in genes that have therapeutic implications. It would be helpful for genetic laboratories to report known diagnostic discordance with other clinical laboratories.

## Key Points

**Question:**

What is the variation in interpretations of genetic test results between laboratories for patients with epilepsy?

**Findings:**

In this cross-sectional study of 22 676 genetic variants associated with epilepsy that were reported to the ClinVar public database, 3.2% of variants interpreted by more than one laboratory had clinically substantial discordance in interpretations.

**Meaning:**

The results of genomic tests performed in patients with epilepsy may receive interpretations that differ based on the laboratory that performed the testing.

## Teaser

This cross-sectional study uses the ClinVar public database to assess interlaboratory variation in the interpretations of genetic test results in patients with epilepsy.

## Introduction

Genomic testing (ie, the simultaneous testing of multiple genes) has become routine in the diagnosis and management of pediatric patients with epilepsy. Genomic testing for epilepsy yields a diagnosis in 20% to 50% of cases.^1,2^ Notably, many genetic forms of epilepsy have specific therapeutic implications.^3^ Previous studies have examined the ways in which interpretations of genetic test results can change with the passage of time and the acquisition of new medical knowledge.^4^ Periodic reinterpretation of genetic variants is recommended.^5,6^ In this context, interlaboratory variation in the interpretations of genetic test results constitutes an additional important source of uncertainty and potential medical error.^7,8^

In this study, we examined the interlaboratory discordance of the interpretation of genetic variants associated with epilepsy. We also focused on the subset of genes associated with epilepsy that have potential therapeutic implications.

## Methods

The data from this study were obtained from a publicly accessible database and contained no patient identifiers. The study was reviewed by the institutional review board of the University of Texas Southwestern Medical Center, which determined that the study did not meet the definition of human subjects research under 45 CFR 46.102 and therefore did not require informed consent. The Strengthening the Reporting of Observational Studies in Epidemiology (STROBE) guideline for cross-sectional studies was used.

Conflicting interpretations of genetic variants associated with epilepsy were analyzed for clinically substantial differences between May 7 and June 29, 2019. A set of 70 genes previously described to be associated with epilepsy were included in the analysis (eTable 1 in the Supplement).^9^ These genes had multiple types of inheritance patterns, including autosomal dominant (n = 31), autosomal recessive (n = 28), and X–linked dominant (n = 4) patterns; 7 genes had multiple inheritance types, including both dominant and recessive associations. A subset of genes with therapeutic implications was also examined.^3^

The ClinVar public database of clinically annotated variants was accessed through the ClinVar Miner search engine (Eilbeck Lab, University of Utah)^10^ using data set version 2019-05. Variant annotations from November 16, 2012, to May 3, 2019, were examined. Variants were examined only if they had been interpreted by 2 or more clinical laboratories. A variant with a clinically substantial difference in interpretation was defined as a variant that crossed the threshold between a likely pathogenic variant and a variant of uncertain significance.

The genes associated with epilepsy were limited to interpretations from clinical laboratories. Conflict types were separated into conflicts of confidence (eg, benign vs likely benign variants and pathogenic vs likely pathogenic variants), benign conflicts (benign or likely benign variants vs variants of uncertain significance), or clinically substantial conflicts (pathogenic or likely pathogenic [pathogenic/likely pathogenic] variants vs benign or likely benign [benign/likely benign] variants and variants of uncertain significance). The category of clinically substantial conflicts implied a difference in interpretations that had implications for the clinical diagnosis and management of a patient. In general, variants of uncertain significance are not clinically actionable and were therefore considered benign conflicts when discordance occurred between the interpretations of a variant of uncertain significance and a benign/likely benign variant. All variants in the category of clinically substantial conflicts in interpretation were further analyzed to identify the types of discrepancies. The period between discrepancies was measured between the most recent submissions in each category (pathogenic/likely pathogenic variants vs benign/likely benign variants and variants of uncertain significance).

### Statistical Analysis

Microsoft Excel software (Microsoft Corp) was used for calculations. Percentages were used for the reporting of descriptive statistics. *P* values were not used for hypothesis testing.

## Results

**Figure 1.**
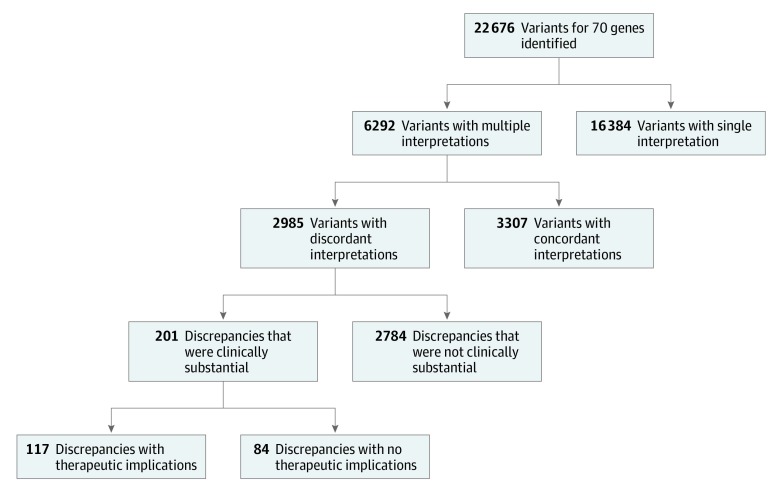
Study Flowchart

**Figure 2.**
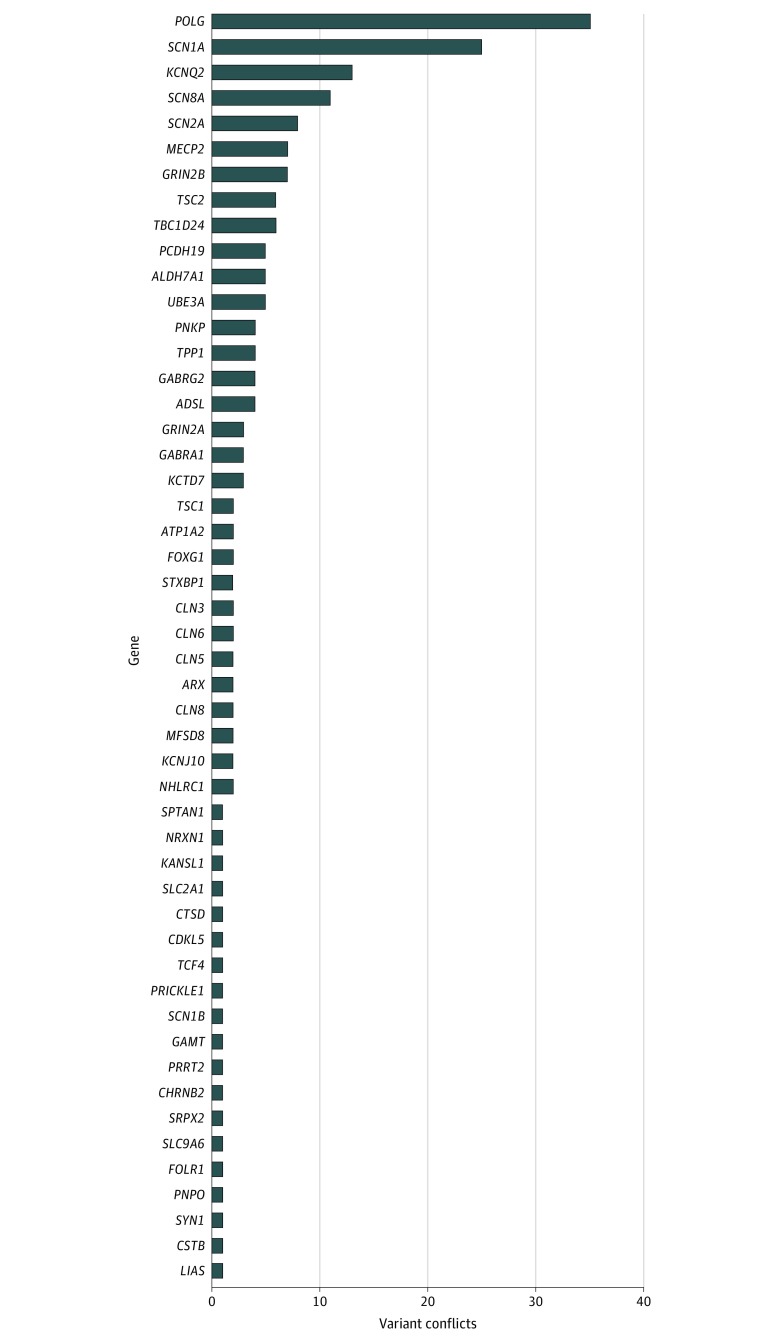
Clinically Substantial Variant Conflicts by Gene

In the 70 genes associated with epilepsy,^9^ 22 676 genetic variants (from an unknown number of patients) with interpretations were present in the ClinVar database. Most variants (16 384 [72.3%]) were interpreted only by a single clinical laboratory, and 6292 variants (27.7%) were interpreted by multiple laboratories (Figure 1). Many variants (3307 of 6292 [52.6%]) had interpretations that were fully concordant. However, 2985 variants (47.4%) had conflicting interpretations. Some variants had multiple conflict types. Most conflicts were minor differences in confidence (benign vs likely benign variants; 1681 of 6292 conflicts [26.7%]) or were nonactionable (benign/likely benign variants vs variants of uncertain significance; 1527 of 6292 conflicts [24.3%]). Interpretations with substantial clinical discordance occurred in 201 of 6292 variants (3.2%) in which a conflict was possible (eTable 2 in the Supplement). Variants with clinically substantial conflicting interpretations occurred in 50 genes. The gene with the highest number of clinically substantial variant conflicts (n = 35) was DNA polymerase gamma (*POLG*; OMIM 174763; Figure 2) (eTable 2 in the Supplement).

**Figure 3.**
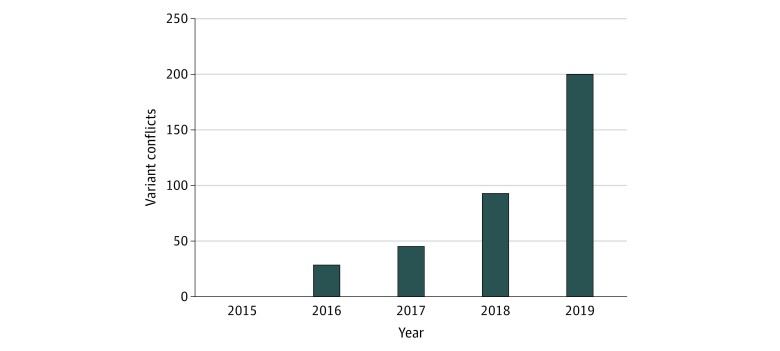
Clinically Substantial Variant Conflicts Over Time The cumulative number of variants with clinically substantial interpretation conflicts that were reported in the ClinVar database increased from 1 to 201 from January 1, 2015, to May 3, 2019. For 2016, 2017, and 2018, the periods examined were January 1 to December 31; for 2019, the period examined was January 1 to May 31.

As a percentage of the total variants in the ClinVar database for a given gene, conflicting variants that were clinically substantial occurred most frequently in the sodium channel voltage-gated type 8 alpha subunit gene, *SCN8A* (OMIM 600702; 11 of 95 variants [11.6%]) and least frequently in the tuberous sclerosis complex subunit 1 gene, *TSC1* (OMIM 605284; 2 of 430 variants [0.5%]). In addition to identifying overall variant interpretation conflicts, we examined the ClinVar data for the number of variant conflicts per year. From January 1, 2015, to May 3, 2019, the number of variants submitted for the 70 genes we examined increased from 1906 to 22 676. During this period, the number of variants with clinically substantial conflicts increased from 1 to 201 (Figure 3).

Of the 201 discrepant variants, only 19 had reported information in ClinVar indicating that a variant was de novo. Twelve of the 19 cases with de novo variants were listed with an interpretation of pathogenic/likely pathogenic.

**Figure 4.**
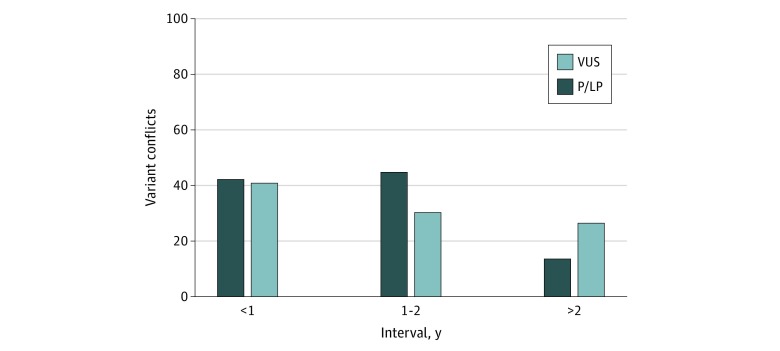
Interval Between Conflicting Interpretations An interval was defined as the elapsed time between 2 conflicting interpretations; when multiple conflicting interpretations existed for a single variant, the 2 most recent conflicting interpretations were used. The total number of variants with conflicting interpretations was 84 in the interval of less than 1 year, 76 in the interval of 1 to 2 years, and 41 in the interval of more than 2 years. P/LP indicates pathogenic or likely pathogenic variants; VUS, variants of uncertain significance.

Variants with clinically substantial conflicts were also examined based on the interval between 2 conflicting interpretations (Figure 4). Three intervals (<1 year, 1-2 years, and >2 years) were used to sort the elapsed time between 2 conflicting interpretations. When multiple conflicting interpretations existed for a single variant, the most recent conflicting interpretation was used. The longest interval (>2 years) had the fewest number of conflicting variants (n = 41) compared with the intervals of less than 1 year (n = 84) and 1 to 2 years (n = 76). When subdivided by the most recent interpretation, pathogenic/likely pathogenic variants were observed in 43 of 84 genes (51.2%) in the interval of less than 1 year, 45 of 76 genes (59.2%) in the interval of 1 to 2 years, and 14 of 41 genes (34.1%) in the interval of more than 2 years.

Of all genes assessed, 117 of the 201 clinically substantial variant conflicts (58.2%) had potential therapeutic implications (eTable 2 in the Supplement). The genes with clinically substantial therapeutic implications were aldehyde dehydrogenase 7 family member A1 (*ALDH7A1*; OMIM 107323), cholinergic receptor neuronal nicotinic beta polypeptide 2 (*CHRNB2*; OMIM 118507), glutamate receptor ionotropic N-methyl-D-aspartate subunit 2A (*GRIN2A*; OMIM 138253), potassium channel voltage-gated subfamily Q member 2 (*KCNQ2*; OMIM 602235), protocadherin 19 (*PCDH19*; OMIM 300460), pyridoxamine 5-prim-phosphate oxidase (*PNPO*; OMIM 603287), proline-rich transmembrane protein 2 (*PRRT2*; OMIM 614386), sodium channel neuronal type 1 alpha subunit (*SCN1A*; OMIM 182389), sodium channel voltage-gated type 2 alpha subunit (*SCN2A*; OMIM 182390), sodium channel voltage-gated type 8 alpha subunit (*SCN8A*; OMIM 600702), solute carrier family 2 member 1 (*SLC2A1*; OMIM 138140), *TSC1* (OMIM 605284), *TSC2* (OMIM 191092), and *POLG*. The gene *SCN1A*, for example, had 25 variants with clinically substantial differences in interlaboratory interpretations (Table). All discrepancies were between the categories of pathogenic/likely pathogenic variants and variants of uncertain significance. An examination of discordant interpretations of the *SCN1A* gene that had minimal clinical implications (eTable 3 in the Supplement) identified 84 variants with varying diagnoses.

**Table.** Clinically Substantial Discordance in Interpretations of *SCN1A* Variants^**a**^

Nucleotide	Amino acid^b^	Lab A	Lab B	Lab C	Lab D	Other^c^
c.472G>C	p.Glu158Gln	LP	VUS	NA	NA	NA
c.602 + 1G>A	NA	P	NA	NA	P	VUS
c.602 + 2dupT	NA	P	VUS	NA	NA	NA
c.791T>C	p.Ile264Thr	LP	NA	NA	VUS	NA
c.986G>T	p.Gly329Val	VUS	NA	NA	NA	LP
c.1216G>T	p.Val406Phe	NA	NA	NA	VUS	P
c.1264G>A	p.Val422Met	NA	NA	NA	LP	VUS
c.2594G>A	p.Arg865Gln	LP	VUS	NA	NA	NA
c.2665G>A	p.Ala889Thr	VUS	LP	NA	NA	NA
c.2729A>G	p.Gln910Arg	LP	VUS	NA	NA	NA
c.2839G>A	p.Val947Met	LP	VUS	NA	NA	NA
c.2941C>A	p.Leu981Ile	LP	NA	NA	NA	VUS
c.3698G>A	p.Gly1233Asp	VUS	NA	NA	NA	LP
c.3776T>C	p.Phe1259Ser	LP	VUS	NA	VUS	NA
c.3879 + 5G>A	NA	LP	NA	NA	NA	VUS
c.4171A>C	p.Asn1391His	LP	VUS	VUS	NA	NA
c.4321G>A	p.Ala1441Thr	LP	VUS	NA	NA	NA
c.4547C>T	p.Ser1516Leu	LP	VUS	NA	NA	NA
c.4556C>T	p.Pro1519Leu	LP	NA	VUS	NA	NA
c.4787G>A	p.Arg1596His	VUS	P	NA	NA	P
c.4793A>T	p.Tyr1598Phe	LP	NA	VUS	VUS	NA
c.4973C>T	p.Thr1658Met	NA	VUS	NA	NA	LP
c.5306A>G	p.Tyr1769Cys	LP	NA	VUS/LP^d^	NA	NA
c.5563C>T	p.Pro1855Ser	LP	NA	NA	VUS	NA
c.5797delC	p.Arg1933Glufs*3	VUS	VUS	NA	NA	P

Abbreviations: LP, likely pathogenic; NA, not applicable; P, pathogenic; VUS, variant of uncertain significance.

^a^ Transcript NM_001165963.2.

^b^ NA indicates splice site variants in which no amino acid was changed.

^c^ Clinical laboratories with 2 or fewer variant conflicts.

^d^ Lab C reported both variants of uncertain significance (RCV000624688.1, 2017-08-04) and likely pathogenic variants (RCV000720726.1, 2017-08-16). NA indicates that no interpretation was submitted by the corresponding laboratory.

## Discussion

We identified 201 variants with clinically substantial differences in variant interpretation among 22 676 variants described in a single database. Most variants were not in conflict because most (72.3%) were only reported by a single laboratory. It is likely that the variants interpreted by only single laboratories would have had some degree of discordance if they had been examined by multiple laboratories.

The number of conflicting variants increased during the 5-year period examined in this study. This increase may have been associated with increased genetic testing and the identification of rare variants by multiple laboratories. A consensus guideline for the interpretation of variants is available^11^; however, differences may exist regarding the ways in which criteria in the guidelines are applied. A previous survey of 9 laboratories identified potential subjectivity in the application of the consensus guidelines.^8^

Of interest, the number of conflicting variants in the longest period between interpretations (>2 years) was lower than that in the 2 shorter periods (<1 year and 1-2 years; Figure 4). Furthermore, the longest period between conflicting interpretations also had the lowest proportion of pathogenic/likely pathogenic variants (34.1%) reported as the most recent interpretation. This finding suggests that, over time, laboratories may be using additional information (eg, population studies) to downgrade the importance of previously reported variants. The shorter periods between conflicting interpretations (<1 year and 1-2 years) had approximately equal proportions of pathogenic/likely pathogenic variants and variants of uncertain significance reported in the most recent interpretation. We believe this finding suggests that the passage of time (>2 years) is more likely to result in downgrading compared with upgrading of the clinical importance of variants. During the shorter periods (<1 year and 1-2 years), laboratories should have had access to similar information, and the conflicts in interpretation may reflect subjectivity in the interpretation of data.

Because monogenic epilepsies can occur sporadically from de novo variant events in genes intolerant to functional variation, we examined the extent to which discrepancies occurred owing to the identification of a de novo variant that was not reported as de novo in the literature. A total of 19 interpretations included information about de novo variants in the ClinVar database submissions. Twelve of these interpretations used the information about a de novo variant to interpret a variant as pathogenic/likely pathogenic, which accounted for a small number of cases (12 of 201 [6.0%]). Patients and physicians should recognize that differences in genetic variant interpretations may occur and can be clinically substantial. In addition to reporting their assessment of a genetic variation, clinical laboratories should also report whether there is known diagnostic uncertainty among clinical laboratories. It may be prudent for clinical teams to use public databases like ClinVar to identify whether conflicts or updates among variant interpretations exist. Notably, even well-studied genes with therapeutic implications, such as *SCN1A*, can have clinically substantial discordance in variant interpretation. Another strategy used to resolve discrepancies in interpretation has been to identify laboratories that have interpretations that are outliers from other clinical laboratories.^12^

To provide context for interlaboratory discrepancies, the practice of surgical pathology can be examined. In the present study, we identified a 3.2% interlaboratory discrepancy; in comparison, studies of second-opinion reviews in the field of surgical pathology have identified diagnostic differences in 1.5% to 6.0% of general cases.^13^ In the subspecialty of hematopathology, 16% of cases have been noted to have diagnostic differences,^14^ with 5% of differences having implications for patient care.^15^ Tertiary care centers routinely perform secondary reviews of surgical pathology cases before recommending definitive therapies, such as surgery, chemotherapy, or radiotherapy. Such a practice of secondary review may be prudent in genomics when a variant is in conflict. A genomic referral for an expert second opinion might be warranted when a management decision about treatment is considered (ie, surgery).

### Limitations

This cross-sectional study had several limitations. The major limitation was that the study was based on a database with variant, but not patient-level, information. Therefore, this study did not examine the discordance in interpretations based on patients but rather on specific variants. A given patient may have had multiple genetic variants identified from a genomic test; each variant may have had differences in interpretation. In addition, without patient-level data, the study could not assess the frequency of single-variant changes in autosomal disorders compared with 2-variant changes in autosomal-recessive disorders. Another limitation of this study was that discordance could have occurred because some laboratories did not update their ClinVar entries. In addition, when we examined the association of de novo variants with the frequency of conflicts, our analysis was limited by the fact that inherited vs de novo status was not consistently reported in the ClinVar database. When de novo status was not reported, it was unclear whether the variant was inherited or whether an evaluation was not performed.

## Conclusions

The current study identified a clinically substantial conflict in genetic variant interpretations in 3.2% of variants associated with epilepsy that were reported in the ClinVar database by more than 1 laboratory. Second opinions for genetic variant interpretations might resolve some discrepancies in variant interpretation. A second-opinion review should result in a clinical report that will become a part of the patient’s medical record, and the patient should be informed that diagnostic uncertainty exists among clinical laboratories. We anticipate that discrepancies in genetic interpretations will increase as genetic testing becomes prominent in the practice of medicine.

## Notes

Supplement. **eTable 1.** Genes Associated With Epilepsy **eTable 2.** Variants With Clinically Substantial Discrepancies **eTable 3.** *SCN1A* Variant Discordance, Not Clinically Substantial
