# Supplementary material for: Assessment of Interlaboratory Variation in the Interpretation of Genomic Test Results in Patients With Epilepsy
Source: JAMA Netw Open. 2020 Apr 29;3(4):e203812. doi: 10.1001/jamanetworkopen.2020.3812 (PMC7191323; doi:10.1001/jamanetworkopen.2020.3812)
Supplement: Supplement. — eTable 1. Genes Associated With Epilepsy eTable 2. Variants With Clinically Substantial Discrepancies eTable 3. SCN1A Variant Discordance, Not Clinically Substantial [file jamanetwopen-3-e203812-s001.pdf]

Supplementary Online Content

SoRelle JA, Pascual JM, Gotway G, Park JY. Assessment of interlaboratory variation in the interpretation of genomic test results in patients with epilepsy. *JAMA Netw Open.* 2020;3(4):e203812. doi:10.1001/jamanetworkopen.2020.3812

eTable 1. Genes Associated With Epilepsy

eTable 2. Variants With Clinically Substantial Discrepancies

eTable 3. *SCN1A* Variant Discordance, Not Clinically Substantial

This supplementary material has been provided by the authors to give readers additional information about their work.

© 2020 SoRelle JA et al. *JAMA Network Open*.

eTable 1. Genes Associated With Epilepsy

ADSL	*CLN5*	*GABRG2*	*LIAS*	*PPT1*	*SRPX2*
ALDH7A1[a]	*CLN6*	*GAMT*	*MAGI2*	*PRICKLE1*	*STXBP1*
ARX	*CLN8*	*GATM*	*MBD5*	*PRRT2*[a]	*SYN1*
ATP1A2	*CNTNAP2*	*GRIN2A*[a]	*MECP2*	*SCARB2*	*TBC1D24*
ATP6AP2	*CSTB*	*GRIN2B*	*MEF2C*	*SCN1A*[a]	*TCF4*
CACNB4	*CTSD*	*GOSR2*	*MFSD8*	*SCN1B*	*TPP1*
CDKL5	*DNAJC5*	*KANSL1*	*NHLRC1*	*SCN2A*[a]	*TSC1*[a]
CHRNA2[a]	*EFHC1*	*KCNJ10*	*NRXN1*	*SCN8A*[a]	*TSC2*[a]
CHRNA4[a]	*EPM2A*[a]	*KCNQ2*[a]	*PCDH19*[a]	*SLC25A22*	*UBE3A*
CHRNA7	*FOLR1*	*KCNQ3*[a]	*PNKP*	*SLC2A1*[a]	*ZEB2*
CHRNB2[a]	*FOXG1*	*KCTD7*	*PNPO*[a]	*SLC9A6*	
CLN3	*GABRA1*	*LGI1*	*POLG*[a]	*SPTAN1*	

[a]Denotes genes with therapeutic significance

© 2020 SoRelle JA et al. *JAMA Network Open*.

eTable 2. Variants With Clinically Substantial Discrepancies

Gene	Nucleotide change	Amino acid change	Transcript	dbSNP
ADSL	c.153+1G>T		NM_000026.2	rs1555903969
ADSL	c.1342T>C	p.Ser448Pro	NM_000026.2	rs771121666
ADSL	c.1400C>G	p.Pro467Arg	NM_000026.2	rs1057521071
ADSL	c.953C>T	p.Pro318Leu	NM_000026.2	rs202064195
ALDH7A1	c.34delG	p.Ala12Leufs	NM_001182.4	rs750693623
ALDH7A1	c.575C>T	p.Thr192Met	NM_001182.4	rs376917645
ALDH7A1	c.664A>G	p.Thr222Ala	NM_001182.4	rs777829351
ALDH7A1	c.1232C>T	p.Pro411Leu	NM_001182.4	rs780233639
ALDH7A1	c.1301A>G	p.Tyr434Cys	NM_001182.4	rs747597620
ARX	c.428_451dup24	p.Ala150_Ala151insGlyAlaAlaAlaAlaAlaAlaAla	NM_139058.2	rs387906493
ARX	c.453_458dupGGCCGC	p.Ala155_Trp156insAlaAla	NM_139058.2	rs398124512
ATP1A2	c.1777C>T	p.Arg593Trp	NM_000702.3	rs886039530
ATP1A2	c.2438T>A	p.Met813Lys	NM_000702.3	rs796052277
CDKL5	c.2842C>T	p.Arg948Ter	NM_001323289.2	rs1555955296
CHRNB2	c.1291G>C	p.Val431Leu	NM_000748.2	rs1064796396
CLN3	c.1213C>T	p.Arg405Trp	NM_001042432.1	rs139842473
CLN3	c.790+3A>C		NM_001042432.1	rs386833738
CLN5	c.1121A>G	p.Tyr374Cys	NM_006493.2	rs148862100
CLN5	c.334C>T	p.Arg112Cys	NM_006493.2	rs786205211
CLN6	c.794_796delCCT	p.Ser265del	NM_017882.2	rs768422260
CLN6	c.898T>C	p.Trp300Arg	NM_017882.2	rs750937323
CLN8	c.209G>A	p.Arg70His	NM_018941.3	rs386834124
CLN8	c.703delC	p.Val236Serfs	NM_018941.3	rs761621368
CSTB	c.1_2insAT	p.Met1Asnfs	NM_000100.3	rs1555955296
CTSD	c.751G>A	p.Asp251Asn	NM_001909.4	rs1064796396
FOLR1	c.493+2T>C		NM_016725.2	rs1044894207
FOXG1	c.670G>A	p.Gly224Ser	NM_005249.4	rs727503935
FOXG1	c.685A>C	p.Ile229Leu	NM_005249.4	rs1064797186
GABRA1	c.335G>A	p.Arg112Gln	NM_000806.5	rs587777308
GABRA1	c.640C>T	p.Arg214Cys	NM_000806.5	rs727503940
GABRA1	c.851T>C	p.Val284Ala	NM_000806.5	rs794727962
GABRG2	c.269C>T	p.Thr90Met	NM000816.3	rs1057520498
GABRG2	c.549-3T>G		NM198903.2	rs750459631
GABRG2	c.1000G>A	p.Ala334Thr	NM000816.3	rs398123523
GABRG2	c.1336C>T	p.Arg446Trp	NM000816.3	rs796052515
GAMT	c.507_521dup	p.Ser173_Trp174insCysAsnLeuThrSer	NM_000156.5	rs763407972
GRIN2A	c.2069C>T	p.Thr690Met	NM_000833.4	rs1445802934
GRIN2A	c.2197G>A	p.Ala733Thr	NM_000833.4	rs796052550
GRIN2A	c.2452G>A	p.Ala818Thr	NM_000833.4	rs1555483699
GRIN2B	c.1547A>G	p.Asn516Ser	NM_000834.4	rs886041295
GRIN2B	c.1672G>A	p.Val558Ile	NM_000834.4	rs1057519004
GRIN2B	c.1727_1732delTCTTTG	p.Val576_Phe577del	NM_000834.4	rs1555111511
GRIN2B	c.2002G>A	p.Asp558Asn	NM_000834.4	rs876661151
GRIN2B	c.2087G>A	p.Arg696His	NM_000834.4	rs1555103971
GRIN2B	c.2450A>G	p.Asn817Ser	NM_000834.4	rs1555103159
GRIN2B	c.2459G>C	p.Gly820Ala	NM_000834.3	rs797044849
KANSL1	c.868C>T	p.Arg290Ter	NM_001193466.1	rs144637717
KCNJ10	c.76C>T	p.Arg26Ter	NM_002241.4	rs138943405
KCNJ10	c.221C>T	p.Thr74Ile	NM_002241.4	rs796052604
KCNQ2	c.593G>A	p.Arg198Gln	NM_172107.3	rs796052621
KCNQ2	c.601C>T	p.Arg201Cys	NM_172107.3	rs796052623
KCNQ2	c.637C>T	p.Arg213Trp	NM_172107.3	rs118192203
KCNQ2	c.701C>T	p.Thr234Ile	NM_172107.3	rs794727741
KCNQ2	c.704C>T	p.Ala235Val	NM_172107.3	rs797045638
KCNQ2	c.782T>C	p.Phe261Ser	NM_172107.3	rs796052631
KCNQ2	c.841G>A	p.Gly281Arg	NM_172107.3	rs794727813
KCNQ2	c.901G>A	p.Gly301Ser	NM_172107.3	rs1057516099
KCNQ2	c.1057C>T	p.Arg353Cys	NM_172107.3	rs118192218
KCNQ2	c.1627G>A	p.Val543Met	NM_172107.3	rs794727134
KCNQ2	c.1639C>T	p.Arg547Trp	NM_172107.3	rs796052650
KCNQ2	c.1887+5G>A		NM_172107.3	rs777916008
KCNQ2	c.2245G>T	p.Glu749Ter	NM_172107.3	rs796052658
KCTD7	c.172G>A	p.Gly58Arg	NM_153033.4	rs750033880
KCTD7	c.190A>G	p.thr64Ala	NM_153033.4	rs201296399
KCTD7	c.456G>A	p.Val152=	NM_153033.4	rs796052686
LIAS	c.983T>A	p.Phe328Tyr	NM_006859.3	rs779931959

© 2020 SoRelle JA et al. *JAMA Network Open*.

MECP2	c.343C>T	p.Arg115Cys	NM_004992.3	rs267608388
MECP2	c.397C>T	p.Arg133Cys	NM_004992.3	rs28934904
MECP2	c.455C>G	p.Pro152Arg	NM_004992.3	rs61748404
MECP2	c.568C>T	p.Arg190Cys	NM_004992.3	rs587783137
MECP2	c.602C>T	p.Ala201Val	NM_004992.3	rs61748381
MECP2	c.1129_1198del70	p.Lys377Profs	NM_004992.3	rs1557135353
MECP2	c.1200A>C	p.Pro400=	NM_001110792.1	rs797044732
MFSD8	c.362A>G	p.Tyr121Cys	NM_152778.2	rs118203978
MFSD8	c.1006G>C	p.Glu336Gln	NM_152778.2	rs150418024
NHLRC1	c.386C>A	p.Pro129His	NM_198586.2	rs750465793
NHLRC1	c.1142A>G	p.Asp381Gly	NM_198586.2	rs200201752
NRXN1	c.3619C>T	p.Arg1207Ter	NM_001135659.2	rs149830411
PCDH19	c.697G>T	p.Asp233Tyr	NM_001184880.1	rs1555985482
PCDH19	c.707C>G	Pro236Arg	NM_001184880.1	rs1060502176
PCDH19	c.790G>T	p.Asp264Tyr	NM_001105243.1	rs587784300
PCDH19	c.1114C>T	p.Arg372Trp	NM_001184880.1	rs796052812
PCDH19	c.1682C>G	p.Pro561Arg	NM_001184880.1	rs796052819
PNKP	c.1029+2T>C		NM_007254.3	rs199919568
PNKP	c.1123G>T	p.Gly375Trp	NM_007254.3	rs786203983
PNKP	c.1385G>C	p.Arg462Pro	NM_007254.3	rs376854895
PNKP	c.968C>T	p.Thr323Met	NM_007254.3	rs372148913
PNPO	c.686G>A	p.Arg229Gln	NM_018129.3	rs773450573
POLG	c.488C>T	p.Pro163Leu	NM_002693.2	rs752892262
POLG	c.752C>T	p.Thr251Ile	NM_002693.2	rs113994094
POLG	c.830A>T	p.His277Leu	NM_002693.2	rs138929605
POLG	c.855G>C	p.Gln285His	NM_002693.2	rs141367015
POLG	c.1156C>T	p.Arg386Cys	NM_002693.2	rs199759055
POLG	c.1276G>A	p.Gly426Ser	NM_002693.2	rs775576189
POLG	c.1402A>G	p.Asn468Asp	NM_002693.2	rs145843073
POLG	c.1760C>T	p.Pro587Leu	NM_002693.2	rs113994096
POLG	c.1763G>A	p.Gly588Asp	NM_002693.2	rs371334941
POLG	c.1790G>A	p.Arg597Gln	NM_002693.2	Chr15(GRCh38):g.89325609G>A
POLG	c.2209G>C	p.Gly737Arg	NM_002693.2	rs121918054
POLG	c.2243G>C	p.Trp748Ser	NM_002693.2	rs113994097
POLG	c.2246T>C	p.Phe749Ser	NM_002693.2	rs202037973
POLG	c.2264A>C	p.Lys755Thr	NM_002693.2	rs770438363
POLG	c.2293C>A	p.Pro765Thr	NM_002693.2	rs1003442806
POLG	c.2419C>T	p.Arg807Cys	NM_002693.2	rs769827124
POLG	c.2606G>A	p.Arg869Gln	NM_002693.2	rs1356604153
POLG	c.2620T>A	p.Leu874Met	NM_002693.2	rs758402960
POLG	c.2636A>G	p.Gln879Arg	NM_002693.2	rs368587966
POLG	c.2642C>T	p.Pro881Leu	NM_002693.2	rs375935084
POLG	c.264C>G	p.Phe88Leu	NM_002693.2	rs144439703
POLG	c.2663G>A	p.Gly888Asp	NM_002693.2	rs878854560
POLG	c.2665G>A	p.Ala889Thr	NM_002693.2	rs763393580
POLG	c.2740A>C	p.Thr914Pro	NM_002693.2	rs139590686
POLG	c.2857C>T	p.Arg953Cys	NM_002693.2	rs11546842
POLG	c.2890C>T	p.Arg964Cys	NM_002693.2	rs201477273
POLG	c.3139C>T	p.Arg1047Trp	NM_002693.2	rs181860632
POLG	c.3151G>A	p.Gly1051Arg	NM_002693.2	Chr15(GRCh38):g.89319053G>A
POLG	c.3151G>C	p.Gly1051Arg	NM_002693.2	rs121918049
POLG	c.3287G>A	p.Arg1096His	NM_002693.2	rs368435864
POLG	c.3383G>A	p.Arg1128His	NM_002693.2	Chr15(GRCh28):g.89318640G>A
POLG	c.3412C>T	p.Arg1138Cys	NM_002693.2	rs767138032
POLG	c.3527C>T	p.Ser1176Leu	NM_002693.2	rs776031396
POLG	c.3573G>T	p.Lys1191Asn	NM_002693.2	rs1085307741
POLG	c.3640C>T	p.Gln1214Ter	NM_002693.2	rs781256643
PRICKLE1	c.824C>T	p.Thr275Met	NM_153026.2	rs199546979
PRRT2	c.922C>T	p.Arg308Cys	NM_145239.2	rs932713001
SCN1A	c.602+1G>A		NM_001165963.2	rs794726827
SCN1A	c.602+2dupT		NM_001165963.2	rs796053054
SCN1A	c.791T>C	p.Ile264Thr	NM_001165963.2	rs745664511
SCN1A	*c.986G>T*	p.Gly329Val	NM_001165963.2	rs779184118
SCN1A	c.1216G>T	p.Val406Phe	NM_001165963.2	rs121918768
SCN1A	c.1264G>A	p.Val422Met	NM_001165963.2	rs886042528
SCN1A	c.2594G>A	p.Arg865Gln	NM_001165963.2	rs1057517862
SCN1A	c.2665G>A	p.Ala889Thr	NM_001165963.2	rs1266877537
SCN1A	c.2729A>G	p.Gln910Arg	NM_001165963.2	rs1064795735
SCN1A	c.2839G>A	p.Val947Met	NM_001165963.2	rs796052986

© 2020 SoRelle JA et al. *JAMA Network Open*.

SCN1A	c.2941C>A	p.Leu981Ile	NM_001165963.2	rs1057518112
SCN1A	c.3698G>A	p.Gly1233Asp	NM_001165963.2	rs1553532470
SCN1A	c.3776T>C	p.Phe1259Ser	NM_001165963.2	rs398123591
SCN1A	c.3879+5G>A		NM_001165963.2	rs796052999
SCN1A	c.4171A>C	p.Asn1391His	NM_001165963.2	rs1295072436
SCN1A	c.4321G>A	p.Ala1441Thr	NM_001165963.2	rs121917974
SCN1A	c.4547C>T	p.Ser1516Leu	NM_001165963.2	rs139300715
SCN1A	c.4556C>T	p.Pro1519Leu	NM_001165963.2	rs372425457
SCN1A	c.472G>C	p.Glu158Gln	NM_001165963.2	rs796053090
SCN1A	c.4787G>A	p.Arg1596His	NM_001165963.2	rs575368466
SCN1A	c.4793A>T	p.Tyr1598Phe	NM_001165963.2	rs377325221
SCN1A	c.4973C>T	p.Thr1658Met	NM_001165963.2	rs121917922
SCN1A	c.5306A>G	p.Tyr1769Cys	NM_001165963.2	rs886039460
SCN1A	c.5563C>T	p.Pro1855Ser	NM_001165963.2	rs794727415
SCN1A	c.5797delC	p.Arg1933Glufs	NM_001165963.2	rs587780446
SCN1B	c.457G>A	p.Asp153Asn	NM_001037.4	rs72550247
SCN2A	c.605C>T	p.Ala202Val	NM_020822.2	rs1553567409
SCN2A	c.1267G>C	p.Val423Leu	NM_020822.2	rs796053180
SCN2A	c.2657T>C	p.Leu886Ser	NM_020822.2	rs796053118
SCN2A	c.4782G>C	p.Trp1594Cys	NM_020822.2	rs1057521747
SCN2A	c.4877G>A	p.Arg1626Gln	NM_020822.2	rs796053155
SCN2A	c.4879G>A	p.Val1627Met	NM_020822.2	rs796053156
SCN2A	c.4886G>A	p.Arg1629His	NM_020822.2	rs796053157
SCN2A	c.5645G>A	p.Arg1882Gln	NM_020822.2	rs794727444
SCN8A	c.632T>C	p.Val211Ala	NM_014191.3	rs1057518487
SCN8A	c.1250A>C	p.Gln417Pro	NM_014191.3	rs878854973
SCN8A	c.1445A>G	p.Lys482Arg	NM_014191.3	rs769520392
SCN8A	c.2549G>A	p.Arg850Gln	NM_014191.3	rs587780586
SCN8A	c.3955G>T	p.Ala1319Ser	NM_014191.3	rs796053214
SCN8A	c.4235T>C	p.Phe1412Ser	NM_014191.3	rs1555228665
SCN8A	c.4441A>G	p.Met1481Val	NM_014191.3	rs886041670
SCN8A	c.4840A>G	p.Thr1614Ala	NM_014191.3	rs1555230909
SCN8A	c.4850G>A	p.Arg1617Gln	NM_014191.3	rs587777721
SCN8A	c.4877G>A	p.Arg1626His	NM_014191.3	rs886044328
SCN8A	c.5630A>G	p.Asn1877Ser	NM_014191.3	rs587780455
SLC2A1	c.400G>A	p.Gly134Ser	NM_006516.3	rs1057518953
SLC9A6	c.430-9_430-5delTTTTA		NM_006359.2	rs796053290
SPTAN1	c.6908_6916dupACCAGCTGG	p.Leu2305_Gly2306insAspGlnLeu	NM_001130438.2	rs587784440
SRPX2	c.1030C>A	p.Leu3444Ile	NM_014467.2	rs149051060
STXBP1	c.734A>G	p.His245Arg	NM_001032221.3	rs587784453
STXBP1	c.874C>T	p.Arg292Cys	NM_003165.3	rs786205598
SYN1	c.1297C>T	p.His433Tyr	NM_133499.2	rs41298474
TBC1D24	c.241_252delATCGTGGGCAAG	p.Ile81_Lys84del	NM_001199107.1	rs761918906
TBC1D24	c.328G>A	p.Gly110Ser	NM_001199107.1	rs747821285
TBC1D24	c.457G>A	p.Glu153Lys	NM_001199107.1	rs376712059
TBC1D24	c.679C>T	p.Arg227Trp	NM_001199107.1	rs748302886
TBC1D24	c.680G>T	p.Arg227Leu	NM_001199107.1	rs756181906
TBC1D24	c.845C>G	p.Pro282Arg	NM_020705.2	rs747538224
TCF4	c.1741G>T	p.Val581Phe	NM_001083962.1	rs587784460
TPP1	c.1016G>A	p.Arg339Gln	NM_000391.3	rs765380155
TPP1	c.1058C>A	p.Thr353Asn	NM_000391.3	rs145966505
TPP1	c.381-10dupT		NM_000391.3	rs146315473
TPP1	c.887-18A>G		NM_000391.3	rs935526225
TSC1	c.737+3A>G		NM_000368.4	rs118203439
TSC1	c.737G>A	p.Arg246Lys	NM_000368.4	rs118203436
TSC2	c.886G>A	p.Val296Met	NM_000548.4	rs747237113
TSC2	c.1864C>T	p.Arg622Trp	NM_000548.4	rs397514914
TSC2	c.2666C>T	p.Ala889Val	NM_000548.4	rs137854155
TSC2	c.3599G>A	p.Arg1200Gln	NM_000548.3	rs397515225
TSC2	c.4747G>A	p.Glu1583Lys	NM_000548.4	rs886039446
TSC2	c.5260-1G>C		NM_000548.3	rs1057518103
UBE3A	c.349T>C	p.Cys117Arg	NM_130838.1	rs587782907
UBE3A	c.947T>C	p.Met316Thr	NM_130838.1	rs863225071
UBE3A	c.1805A>G	p.Asn602Ser	NM_130838.1	rs587784521
UBE3A	c.2503_2508delCTTAAA	p.Leu835_Lys836del	NM_130838.1	rs863225070
UBE3A	c.2503C>T	p.Leu835Phe	NM_130838.1	rs587783097

© 2020 SoRelle JA et al. *JAMA Network Open*.

eTable 3. *SCN1A* Variant Discordance, Not Clinically Substantial

Nucleotide change	Amino acid change	Transcript	Lab A	Lab B	Lab C	Lab D	Others
c.2T>C	p.Met1Thr	NM_001165963.2	P	LP			
c.68C>T	p.Ala23Val	NM_006920.6	B	LB			
c.90A>G	p.Ala30=	NM_001165963.1	LB			VUS	
c.144C>T	p.Gly48=	NM_001165963.1	LB			VUS	
c.251A>G	p.Tyr84Cys	NM_006920.4	P	LP			
c.265-4A>G		NM_001165963.1	B	LB			
c.333A>G	p.Leu111=	NM_001165963.1	B				VUS
c.345T>C	p.Asn115=	NM_001165963.1		B	B	B	LB
c.579C>T	p.Leu193=	NM_001165963.1	B			VUS	
c.694+10A>G		NM_001165963.1	B	LB		VUS	
c.852T>C	p.Asn284=	NM_001165963.1	LB			VUS	
c.965-1G>A		NM_001165963.1	P	LP			
c.1000C>G	p.Leu334Val	NM_001165963.1	LB	VUS	LB		VUS
c.1065T>C	p.Gly355=	NM_001165963.1	B				LB
c.1131A>C	p.Arg377=	NM_001165963.1	B	B	LB	B	LB, VUS
c.1150T>A	p.Trp384Arg	NM_001165963.1	LP	P			
c.1171-10_1171-9delTT		NM_001165963.1	B	B		B	LB
c.1212A>G	p.Val404=	NM_001165963.1			B	B	B
c.1329A>G	p.Glu443=	NM_001165963.2		LB			
c.1378-3T>C		NM_001165963.1	B	LB	LB		
c.1410C>T	p.Ser470=	NM_001165963.1	B		LB		VUS
c.1499G>A	p.Arg500Gln	NM_001165963.1	VUS	LB		VUS	
c.1625G>A	p.Arg542Gln	NM_006920.4	B	B	LB	LB	VUS
c.1662+9C>A		NM_001165963.1	B	B		B	LB
c.1680T>C	p.Arg560=	NM_001165963.2		LB	LB	VUS	
c.1739G>A	p.Arg580Gln	NM_001165963.1	VUS	VUS		VUS	LB
c.1797G>A	p.Glu599=	NM_001165963.2		LB	LB	VUS	
c.1803C>T	p.Asn601=	NM_001165963.2		LB			VUS
c.1811G>A	p.Arg604His	NM_006920.4	B	B	B	B	LB
c.1818T>C	p.Asp606=	NM_001165963.1		LB		VUS	
c.2044-5delT		NM_001165963.1	LB	B			LB
c.2044-5dupT		NM_001165963.1	B	B		VUS	
c.2176+11A>C		NM_001165963.1	LB				VUS
c.2292T>C	p.Val764=	NM_001165963.1			B	B	LB, B
c.2378C>T	p.Thr793Met	NM_001165963.1	VUS	LB			
c.2421C>T	p.Phe807=	NM_001165963.1	B	B	LB	LB	LB
c.2590C>T	p.Leu864=	NM_001165963.2	LB	VUS			
c.2591C>T	p.Thr864Met	NM_006920.4		P	LP		
c.2758C>T	p.Arg920Cys	NM_006920.4	P	P		LP	
c.2889T>C	p.Ala963=	NM_001165963.1		B	LB	B	LB
c.3039A>G	p.Gln1013=	NM_001165963.2		VUS	LB		VUS
c.3060C>A	p.His1020Gln	NM_001165963.1	VUS	LB		VUS	
c.3199G>A	p.Ala1067Thr	NM_001165963.1	B		B	B	B, LB
c.3481G>A	p.Ala1161Thr	NM_001165963.1	VUS	LB		VUS	
c.3488C>G	P.Thr1163Ser	NM_001165963.1	LB	B	LB	VUS	LB
c.3573T>C	p.Cys1191=	NM_001165963.1	LB	LB			VUS
c.3585T>C	p.As1195=	NM_001165963.1	LB			VUS	
c.3591A>G	p.Glu1197=	NM_001165963.2	LB			VUS	
c.3681A>C	p.Glu1227Asp	NM_006920.4	VUS	LB		VUS	

© 2020 SoRelle JA et al. *JAMA Network Open*.

c.3690T>C	p.Tyr1230=	NM_006920.4	B	LB	B	B	LB
c.3701G>A	p.Arg1234Gln	NM_006920.4	P	LP			
c.3705+10delA		NM_001165963.1	B				VUS
c.3749C>T	p.Thr1250Met	NM_001165963.1	VUS	LB			
c.3886T>C	p.Leu1296=	NM_001165963.1	B	B	LB		
c.3891A>T	p.Glu1297Asp	NM_006920.4	LB	LB	LB	VUS	
c.3899C>T	p.Thr1300Ile	NM_001165963.1	VUS	VUS	LB	VUS	
c.3948G>A	p.Arg1316=	NM_001165963.1	B	B	LB	LB	
c.4129A>G	p.Il31377Val	NM_001165963.1	VUS		LB	VUS	
c.4167C>T	p.Asp1389=	NM_001165963.1	LB	LB			VUS
c.4339-5G>A		NM_001165963.1		LB		VUS	
c.4393A>G	p.Ile1465Val	NM_001165963.1	B	LB	LB	B	
c.4548G>A	p.Ser1516=	NM_001165963.1	B	B	LB	B	
c.4551A>G	p.Ly1517=	NM_001165963.1	LB	B	LB		LB
c.4557G>A	p.Pro1519=	NM_001165963.1	B	LB	LB		
c.4581+12C>T		NM_001165963.1	LB				VUS
c.4724G>A	p.Arg1575His	NM_001165963.1	VUS	LB		VUS	
c.4729T>C	p.Cys1577Arg	NM_006920.4	LP	P			
c.4731T>C	p.Asn1577=	NM_001165963.1	B	B	LB	LB	VUS
c.4789G>T	p.Asp1597Tyr	NM_006920.4		LP			P
c.4855A>G	p.Met1619Val	NM_001165963.1	VUS	B			
c.4872G>A	p.Leu1624=	NM_001165963.1	B	B	LB	VUS	
c.4887C>T	p.Phe1629=	NM_001165963.1	LB				VUS, LB
c.4905C>T	p.Phe1635=	NM_001165963.2	LB				B
c.4945C>T	p.Leu1649=	NM_001165963.1	B	B	LB	B	LB
c.5217C>T	p.Pro1739=	NM_001165963.2		LB			VUS
c.5286A>G	p.Gly1762=	NM_001165963.1		B	LB	LB	
c.5346C>T	p.Ile1782=	NM_001165963.1	B				VUS
c.5418G>A	p.Glu1806=	NM_001165963.1	B	B	B	B	LB
c.5532A>G	p.Pro1844=	NM_001165963.1	LB			VUS	
c.5568G>A	p.Met1856Ile	NM_001165963.1	VUS	LB	VUS	VUS	
c.5693C>T	p.Thr1898Ile	NM_006920.4	LP	P			
c.5749C>G	p.Arg1917Gly	NM_006920.4	LB	B	LB	LB	LB
c.5809C>G	p.Gln1937Glu	NM_001165963.2	VUS	LB			
c.5864T>C	p.Ile1955Thr	NM_001165963.1	B	LB	B	B	LB

SCN1A Transcript NM_001165963.2. [a] An empty cell is present for splice site variants as no amino acid is changed. Empty cells under the "Lab" categories are present when no interpretation was submitted from the corresponding laboratory. P, pathogenic; LP, likely pathogenic; VUS, variant of uncertain significance; LB, likely benign; B, benign.

© 2020 SoRelle JA et al. *JAMA Network Open*.
